# Constitutional Infection Due to Chronic Dento-Alveolar Abscess and Pyorrhea Alveolaris

**Published:** 1915-10

**Authors:** L. W. Doxtater

**Affiliations:** New York, N. Y.


### CONSTITUTIONAL INFECTION DUE TO CHRONIC DENTO-ALVEOLAR ABSCESS AND PYORRHEA ALVEOLARIS.

BY L. W. DOXTATER, D.D.S., NEW YORK, N. Y.

It is important for the dental practitioner to recognize the constitutional diseases due to local infections in the oral cavity.

It is only recently that we have begun to appreciate how common is the contamination of the blood by living organisms. For some time only the more serious conditions of septicemia and pyemia were recognized as being caused by living bacteria in the blood, whereas this fluid may contain many living bacteria that may be a source of disease, such as streptococci, staphylococci, and various bacilli.

ORAL INFECTION AS THE CAUSE OF SYSTEMIC DISEASE.

The leading physicians of the country today first look to the oral cavity for infections that may be the causative factors in systemic diseases. Murphy states that every type of non-traumatic inflammation of the joint is the metastatic manifestation of a primary infection in some other part of the body.

Dentists are becoming cognizant of the fact that a large percentage of infectious arthritic conditions is due to pyorrhea alveolaris. Pyorrhea accounts for an enormous amount of blood infection; 75 per cent. of these cases can be healed by treatment, and most of the others can be held in check by continued treatment. In some cases, removal of the teeth is necessary to accomplish a cure. Removing teeth that are hoplessly infected prevents the harboring of certain bacteria.

The nasal cavity has its special group of bacterial flora, but the great majority of pyogenic micro-organisms affecting the body must enter through the mouth.

In the mouth the teeth are subject to infective destruction from many causes, but by far the most destructive are root abscesses that are developed from diseased pulps of teeth. Very frequently these abscesses are not suspected until their presence is revealed by the X-ray.

PRACTICAL CASES.

The following are a few cases from my private practice:

*Case I.* Mr. B., thirty-seven years. The patient had been suffering from severe pains on the right side of the face and rheumatic pains in the right shoulder; had been treated by three specialists without result. He then consulted Dr. Thomas H. Russell, who referred the case to me.

The radiograph revealed an abscessed upper right canine with perforated root, and a perforated lower right first molar with putrescent root-canals. Both roots were extracted, and the patient has had no trouble since.

*Case II.* Miss S., twenty-eight years. The patient had been suffering from periodic pains in the lower jaw, at times excruciating, and had been treated for neuralgia without any benefit whatever. The case was then referred to me. The lower jaw was radiographed and revealed an impacted bicuspid. The tooth was removed, and the patient experienced no further trouble.

*Case III.* Mr. B., fifty-eight years. The patient presented a very severe case of pyorrhea alveolaris arthritis, with fingers and elbow-joints badly swollen. His breath was fetid, the teeth were loose, and discharging pus; there was also slight fever. The teeth were cleaned, and all pus pockets cleansed by pressure and irrigation with solution of echinacea, followed by a saline solution. The gums were then painted with tincture of iodin. These measures were adopted to eliminate as fully as possible all pus exudate; then, by means of a blunt-pointed hypodermic syringe, a few drops of a one per cent. solution of emetin hydrochlorid were forced into the pus pockets, and their margins anointed with vaselin to retain the solution in contact with the pocket walls as long as possible. This treatment was repeated every other day until all pus flow and inflammation subsided, when thorough instrumentation for the removal of deposits from the root surfaces was instituted. At the end of the fifth week, the systemic conditions had entirely disappeared.

*Case IV.* Mr. B., sixty-four years. In 1859 the patient cut the anterior chamber of the right eye with the point of a knife. The fluid ran out, producing total blindness in that eye. At first the organ gave the patient no trouble, but by 1878 it had become inflamed, with calcareous formation at the back, and was removed.

The left eye was somewhat affected before the operation, but in a few weeks regained its strength, and gave no further trouble. In 1896 the upper left canine was cut off and a Richmond crown inserted, to which was attached a substitute for the adjoining incisor, which had been extracted some time before.

About 1901 the left eye became subject to aches of a few hours' duration, and began to feel weak, with the pain recurring more frequently and remaining longer, accompanied with an overflow of tears. The patient then consulted an oculist, who found conjunctivitis on the lower lid with a trace on the upper one. As time passed, the symptoms became aggravated. Treatments were continued by the oculist at intervals until 1907. The redness on the lower lid persisted, although every remedy known to the profession had been applied, and it was deemed best to discontinue the treatment.

A few years later the patient consulted another oculist, but obtained no relief. He found nothing except the conjunctivitis, which he was sure could not produce the trouble.

In 1914 the slight pain and overflow were present about one-third of the time, at irregular intervals, and gave the patient great discomfort and considerable anxiety. He again consulted the second oculist, who found the duct open and the ball of the eye normal. In his opinion the conjunctivitis was too slight to cause the trouble complained of.

The patient then consulted me as to the possible effect upon the eye of the crowned canine, which for several years had given evidence of slight irregularity, though not of a character to be called pain. The radiograph revealed a blind abscess at the root of the canine. The end of the root was amputated, but the affection of the bone was too great for recovery, so the tooth was extracted.

This operation was performed in January, 1915. Gradually the troublesome conditions disappeared, and from January until now the patient has had his first enjoyment of life in many years.

*Case V.* Mrs. G., forty-one years. The patient, whose mouth was in very bad condition, had been confined to bed because of swelling in her knee-joints. I diagnosed the case as pyorrhea alveolaris arthritis. Several teeth were extracted, and the gums and remaining teeth were treated. At the end of ten days the patient was very much improved, and at the end of the third week all the inflammation left the joints, and the patient seemed to be in the best of health.

*Case VI.* Mrs. M., thirty-six years. . When the patient came under my observation, she presented a very severe case of pyorrhea alveolaris arthritis. Her fingers, knees, and elbow-joints were involved. Raising the forearm above the head caused severe pain. An examination of the oral cavity showed several ill-fitting crowns and two-ill-fitting bridges. The bridges and crowns were removed, and most of the teeth responded to treatment, but it was necessary to extract two lower molars. The pyorrhea was arrested, the teeth tightened firmly, and new bridge work inserted, and the systemic conditions cleared up.

Dr. C. H. Mayo, in a recent article, made the following statement: "It falls upon the dentist and oral surgeon to study the disease conditions of the mouth. Dental literature is full of it, and much original work has been done by such leaders as Black, Talbot, Nodine, Hartzell, Brophy, and numerous others. The work is discouraging, but must be kept up, as eventually it will have its effect. The dentist's patients must be warned of the mouth as being by far the greatest portal of entrance of germ life into the body, the most infected part of the alimentary canal. The people will gradually demand more of their medical advisers. The next great step in medical progress in the line of preventive medicine should be made by the dentist."—*Cosmos.*

